# Deriving the Bidomain Model of Cardiac Electrophysiology From a Cell-Based Model; Properties and Comparisons

**DOI:** 10.3389/fphys.2021.811029

**Published:** 2022-01-07

**Authors:** Karoline Horgmo Jæger, Aslak Tveito

**Affiliations:** ^1^Simula Research Laboratory, Oslo, Norway; ^2^Department of Informatics, University of Oslo, Oslo, Norway

**Keywords:** bidomain model, EMI model, cell-based model, cardiac electrophysiology, cardiac conduction, cardiac tissue models, numerical simulation

## Abstract

The bidomain model is considered to be the gold standard for numerical simulation of the electrophysiology of cardiac tissue. The model provides important insights into the conduction properties of the electrochemical wave traversing the cardiac muscle in every heartbeat. However, in normal resolution, the model represents the average over a large number of cardiomyocytes, and more accurate models based on representations of all individual cells have therefore been introduced in order to gain insight into the conduction properties close to the myocytes. The more accurate model considered here is referred to as the EMI model since both the extracellular space (E), the cell membrane (M) and the intracellular space (I) are explicitly represented in the model. Here, we show that the bidomain model can be derived from the cell-based EMI model and we thus reveal the close relation between the two models, and obtain an indication of the error introduced in the approximation. Also, we present numerical simulations comparing the results of the two models and thereby highlight both similarities and differences between the models. We observe that the deviations between the solutions of the models become larger for larger cell sizes. Furthermore, we observe that the bidomain model provides solutions that are very similar to the EMI model when conductive properties of the tissue are in the normal range, but large deviations are present when the resistance between cardiomyocytes is increased.

## 1. Introduction

Mathematical models are indispensable for understanding the complex processes underlying cardiac electrophysiology. A wide variety of models have been developed for the key processes going on across the membrane of cardiomyocytes (see, e.g., Rudy and Silva, 2006; Rudy, 2012; Qu et al., 2014; Amuzescu et al., 2021), where the latter paper presents a comprehensive overview of the evolution of these models. The models of the membrane dynamics have also been extended to yield descriptions of the electrophysiological properties of cardiac tissue, commonly represented by the bidomain model or the somewhat simpler monodomain model (see Tung, 1978; Neu and Krassowska, 1993; Sundnes et al., 2007; Clayton and Panfilov, 2008; Vigmond et al., 2008; Linge et al., 2009; Niederer et al., 2011a; Franzone et al., 2014). The use of mathematical models for understanding the properties of the cardiac action potential (AP) across the membrane of cardiomyocytes is very widespread, and so is the use of the bidomain/monodomain models for understanding the properties of the excitation wave traversing cardiac tissue during each heartbeat. However, the spatial bidomain/monodomain models have two inherent limitations. The main limitation is that the extracellular space, the membrane of the myocyte, and the intracellular space are all assumed to be present everywhere. This assumption is indeed courageous but has provided surprisingly accurate results and presently underpins the understanding of cardiac conduction. The second limitation is that convergence is obtained using a relatively coarse mesh (Δ*x* ~ 0.25 mm, see Xie et al., 2004; Clayton and Panfilov, 2008; Niederer et al., 2011b) and thus a typical mesh block contains several hundred cardiomyocytes (see, e.g., Jæger et al., 2021a,b). Therefore, understanding of the conduction properties (see, e.g., Henriquez, 2014; Veeraraghavan et al., 2014) close to the myocytes cannot be achieved using these models (see, e.g., Jæger et al., 2021a).

These limitations of the homogenized (bidomain/monodomain) models are well known and several authors have developed alternatives where all individual cells are explicitly represented in the models (see, e.g., Spach et al., 2007; Jacquemet and Henriquez, 2009; Hubbard and Henriquez, 2014; Lin and Keener, 2014; Tveito et al., 2017a; Weinberg, 2017; Jæger et al., 2019, 2021a; Domínguez et al., 2021; Jæger and Tveito, 2021). Here, we will apply the EMI model where both the extracellular space (E), the cell membrane (M) and the intracellular space (I) are explicitly represented in the model (see, e.g., Tveito et al., 2017a,b; Jæger and Tveito, 2021), and compare properties with the homogenized bidomain model. First, we will show how the bidomain model can be derived from the more accurate EMI model. Earlier derivations of the bidomain equations (see, e.g., Neu and Krassowska, 1993; Franzone et al., 2014; Henriquez and Ying, 2021) relies on homogenization of cardiac tissue, whereas the derivation given here follows directly from the EMI model. As part of this derivation, we can identify the main sources of deviations between the models.

Next, we will compare the properties of the bidomain model and the EMI model using numerical simulations. We first show that the deviations between the results obtained by the bidomain model and the EMI model become small as the cell size is reduced. This property is consistent with the error introduced in the derivation of the bidomain model. Secondly, we demonstrate that, for conduction properties providing a normal excitation wave with a conduction velocity of about 50 cm/s, the solutions of the EMI model and the bidomain model are very similar. However, as the resistance between the myocytes (through the gap junctions) is increased, the deviation between the solutions increases considerably.

It should be noted that the representation of all individual cardiomyocytes implies a significant increase in the computation load since the mesh resolution needs to be reduced from about Δ*x* ~ 0.25 mm for a finite difference method (FDM) of the bidomain model to about δ*x* ~ 10 μm for a finite element method (FEM) code solving the EMI model (see Jæger et al., 2021a,b). The number of mesh blocks is Δ*x*^3^/δ*x*^3^ = 15, 600 times larger for the EMI model than for the bidomain model, and, therefore, the computational load increases significantly when every myocyte in the tissue is resolved.

The choice of using either an averaged model like the bidomain model or a cell-based model like the EMI model, depends on the application under consideration. The bidomain model is very useful for simulating large scale problems, whereas EMI is better suited when the dynamics close to individual myocytes, or even inside individual myocytes, are of importance.

## 2. Methods

In this section we will derive the bidomain model commonly used to model the electrical activity of the heart from a more detailed model where each cell is represented. This cell-based model is referred to as the EMI model and is derived from Maxwell's equations of electromagnetism in Agudelo-Toro (2012) and Jæger and Tveito (2021). We will start by introducing the equations of the EMI model before we describe the derivation of the bidomain model from these equations. Finally, we discuss how the bidomain model parameters can be defined using the parameter values and tissue geometry of the EMI model.

### 2.1. The EMI Model

Consider a domain consisting of a single cell, Ω_*i*_, surrounded by an extracellular space, Ω_*e*_, with a cell membrane, Γ, separating the two spaces Ω_*i*_ and Ω_*e*_. For such a domain, the electrical activity may be modeled by the EMI model (see, e.g., Roberts et al., 2008; Stinstra et al., 2010; Tveito et al., 2017a; Jæger et al., 2019), given by the equations


(1) $$\begin{array}{l} \;\nabla \cdot \sigma_{i}\nabla u_{i}=0,\text{ in}\Omega_{i} \end{array}$$

(2) $$\begin{array}{l} \;\nabla \cdot \sigma_{e}\nabla u_{e}=0,\text{ in}\Omega_{e} \end{array}$$

(3) $$\begin{array}{l} {\text{n}}_{e}\cdot \sigma_{e}\nabla u_{e}=-{\text{n}}_{i}\cdot \sigma_{i}\nabla u_{i}\equiv I_{m},\text{ at }\Gamma , \end{array}$$

(4) $$\begin{array}{l} \;u_{i}-u_{e}=v\text{ at }\Gamma , \end{array}$$

(5) $$\begin{array}{l} \;I_{m}=C_{m}\frac{\partial v}{\partial t}+I_{\text{ion}}\text{ at }\Gamma , \end{array}$$

(6) $$\begin{array}{l} \;u_{e}=0\text{ at }\partial \Omega_{e}^{D}, \end{array}$$

(7) $$\begin{array}{l} \;\frac{\partial u_{e}}{\partial {\text{n}}_{e}}=0\text{ at }\partial \Omega_{e}^{N}. \end{array}$$

Here, *u*_*i*_, *u*_*e*_, and *v* are the intracellular, extracellular and membrane potentials (in mV) defined in Ω_*i*_, Ω_*e*_ and at Γ, respectively, **n**_*i*_ and **n**_*e*_ are the outward pointing normal vectors of the intracellular and extracellular spaces, respectively, *C*_*m*_ is the specific membrane capacitance (in μF/cm^2^), *I*_ion_ is the ionic current density across the membrane (in μA/cm^2^), *I*_*m*_ the sum of the capacitive and ionic current densities (in μA/cm^2^), and σ_*i*_ and σ_*e*_ are the intracellular and extracellular conductivities, respectively (in mS/cm). The Equations (6) and (7) are Dirichlet and Neumann boundary conditions, respectively, for the outer boundary of the extracellular space.

#### 2.1.1. Extension to Cells Connected by Gap Junctions

**Figure 1:**
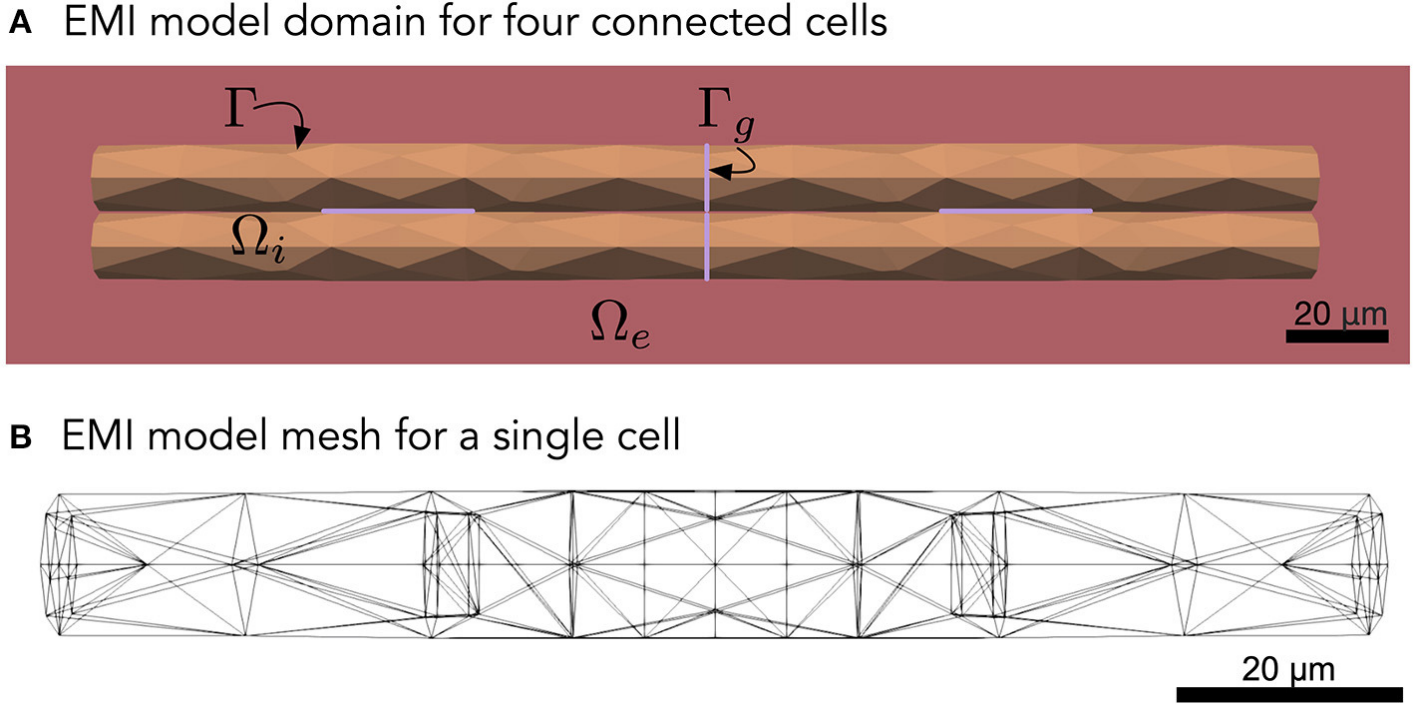
**(A)** Illustration of an EMI model domain for four connected cells. The intracellular space (orange) is denoted by Ω_*i*_ and the extracellular space (red) is denoted by Ω_*e*_. The cell membrane is defined as the interface between the intracellular and extracellular spaces and is denoted by Γ. Similarly, the intercalated discs (purple) are denoted by Γ_*g*_ and are defined as the interface between neighboring cells. Each cell is shaped as a cylinder with a diameter increasing slightly toward the center of the cell. **(B)** Illustration of the finite element mesh used to represent a single cell in the EMI model simulations.

To model collections of connected cardiac cells, e.g., like illustrated in Figure 1A, the EMI model for a single cell may be extended to include a model for the currents through the gap junctions connecting neighboring cells (see, e.g., Tveito et al., 2017a; Jæger and Tveito, 2021; Jæger et al., 2021c). For example, for two connected cells 1 and 2, the EMI model can be extended to include equations of the form


(8) $$\begin{array}{l} \;{\text{n}}_{i}^{2}\cdot \sigma_{i}\nabla u_{i}^{2}=-{\text{n}}_{i}^{1}\cdot \sigma_{i}\nabla u_{i}^{1}\equiv I_{1,2},\text{ at }\Gamma_{g}, \end{array}$$

(9) $$\begin{array}{l} \;u_{i}^{1}-u_{i}^{2}=w,\text{ at }\Gamma_{g}, \end{array}$$

(10) $$\begin{array}{l} I_{1,2}=C_{g}\frac{\partial w}{\partial t}+I_{\text{gap}},\text{ at }\Gamma_{g}, \end{array}$$

where Γ_*g*_ is the interface between the two cells (i.e., the intercalated disc). Furthermore, $u_{i}^{1}$ and $u_{i}^{2}$ are the intracellular potentials of the two cells, *w* is the potential difference between the two cells, and ${\text{n}}_{i}^{1}$, and ${\text{n}}_{i}^{2}$ are the outward pointing normal vectors of the cells. In addition, *C*_*g*_ is the specific capacitance of the intercalated discs (in μF/cm^2^), *I*_gap_ is the current density through the gap junction proteins located at the intercalated discs (in μA/cm^2^), and *I*_1,2_ is the sum of the capacitive current density over the intercalated discs and the current density through the gap junction proteins connecting the two cells. The current density through the gap junction proteins, *I*_gap_, is commonly modeled using the simple passive model


(11) $$\begin{array}{l} I_{\text{gap}}=\frac{1}{R_{g}}w=G_{g}w, \end{array}$$

where *R*_*g*_ is the specific resistance of the gap junctions (in kΩcm^2^) and *G*_*g*_ is the corresponding specific conductance (in mS/cm^2^). A further explanation of the coupling between two adjacent cells is given in section 1.2.4 of Jæger and Tveito (2021).

### 2.2. Derivation of the Bidomain Model From the EMI Model

Instead of using the detailed model (Equations 1–11), modeling of the electrical activity of cardiac tissue is usually performed using the homogenized bidomain and monodomain models. In these models, the detailed geometry of the individual cells and intercalated discs do not have to be represented in the computational mesh because the intracellular space, the extracellular space and the cell membrane are all assumed to exist everywhere in the tissue. We will now describe a possible derivation of the homogenized bidomain model from the EMI model equations described above. Note, however, that more rigorous versions of this derivation, using mathematical two-scale homogenization, have also been presented (see, e.g., Neu and Krassowska, 1993; Franzone et al., 2014; Henriquez and Ying, 2021).

#### 2.2.1. Starting Point of the Derivation

**Figure 2:**
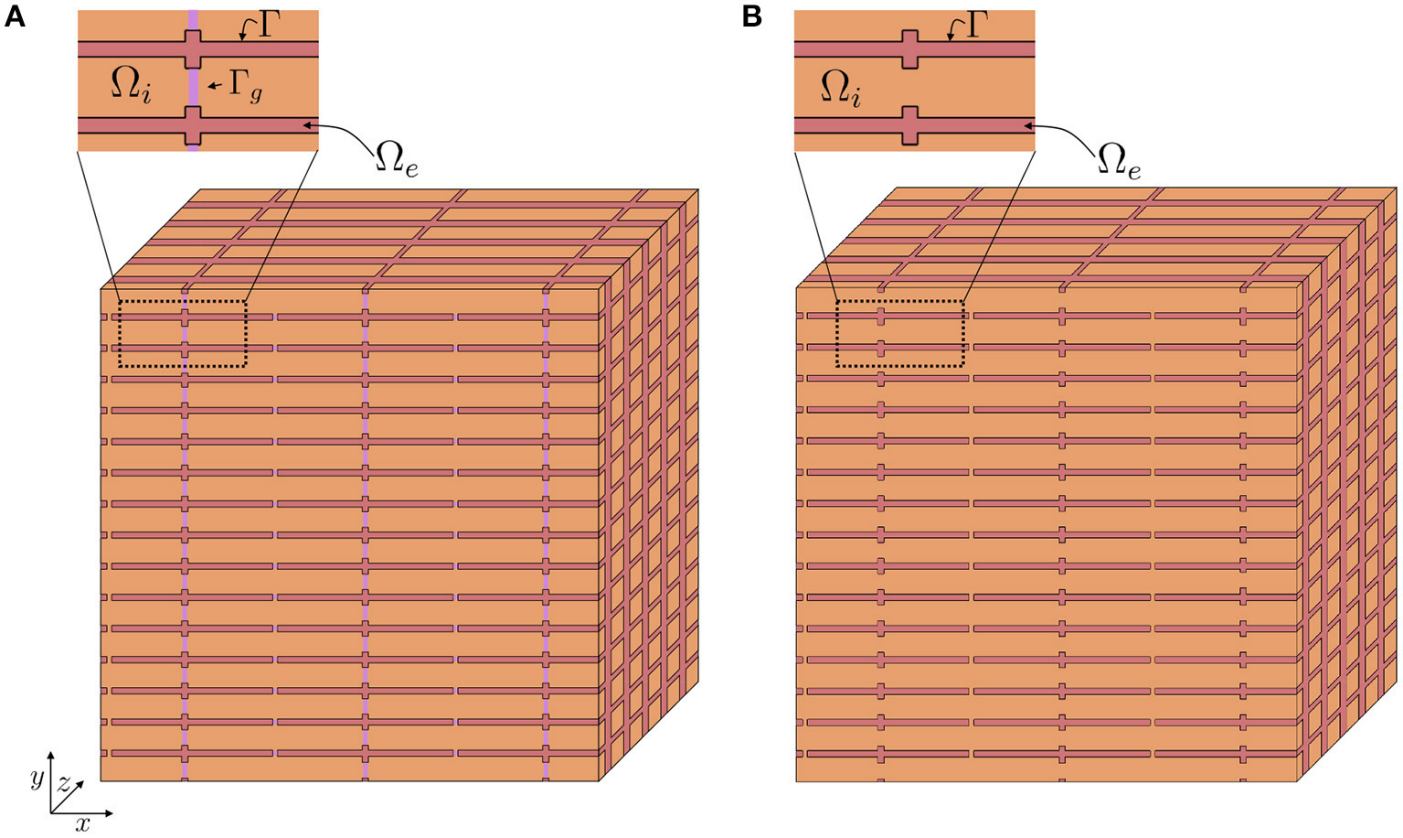
Illustration of a tissue block, Δ, containing a number of cells (orange) and a surrounding extracellular space (red). In Step 1 of the derivation we approximate the discontinuous intracellular space **(A)** consisting of individual cells connected by gap junctions by a continuous intracellular space **(B)**.

Assume that we have a relatively large collection of cells, and consider a small volume, Δ, in this cell collection, as illustrated in Figure 2A. We assume that this volume contains a number of cells with an associated surrounding extracellular space, and that the EMI model equations apply in the extracellular domain, in the intracellular domain, at the cell membrane and at the intercalated discs in this small block of tissue.

##### Step 1: Approximating the Intracellular Conductivity

As a first step in the derivation, we wish to approximate the intracellular conductivity to take both the purely intracellular space and the gap junctions between neighboring cells into account. In other words, we wish to reformulate the full EMI model (Equations 1–11) to a system of the form


(12) $$\begin{array}{l} \;\nabla \cdot {\overset{-}{\sigma }}_{i}\nabla u_{i}=0,\text{ in}\Omega_{i} \end{array}$$

(13) $$\begin{array}{l} \;\nabla \cdot \sigma_{e}\nabla u_{e}=0,\text{ in}\Omega_{e} \end{array}$$

(14) $$\begin{array}{l} {\text{n}}_{e}\cdot \sigma_{e}\nabla u_{e}=-{\text{n}}_{i}\cdot {\overset{-}{\sigma }}_{i}\nabla u_{i}\equiv I_{m},\text{ at }\Gamma , \end{array}$$

(15) $$\begin{array}{l} \;u_{i}-u_{e}=v\text{ at }\Gamma , \end{array}$$

(16) $$\begin{array}{l} \;I_{m}=C_{m}\frac{\partial v}{\partial t}+I_{\text{ion}}\text{ at }\Gamma , \end{array}$$

(17) $$\begin{array}{l} \;u_{e}=0\text{ at }\partial \Omega_{e}^{D}, \end{array}$$

(18) $$\begin{array}{l} \;\frac{\partial u_{e}}{\partial {\text{n}}_{e}}=0\text{ at }\partial \Omega_{e}^{N}, \end{array}$$

where ${\overset{-}{\sigma }}_{i}$ is the average conductivity of the intracellular space including gap junctions. We wish to express ${\overset{-}{\sigma }}_{i}$ such that the total intracellular resistance of a tissue block is close to the total intracellular resistance of the tissue block when the full EMI model (Equations 1–11) applies. Such an expression for ${\overset{-}{\sigma }}_{i}$ is derived below in section 2.3.3.

In the remaining part of the derivation we will treat the intracellular domain as a continuous domain (see Figure 2B), and assume that the simplified EMI system (Equations 12–18) applies.

##### Step 2: Applying the Divergence Theorem

In the next step of the derivation, we consider the purely intracellular part of the tissue block Δ and apply the divergence theorem for ${\overset{-}{\sigma }}_{i}\nabla u_{i}$ to obtain,


(19) $$\begin{array}{l} \int_{\partial \Omega_{i}^{\Delta }}{\text{n}}_{i}\cdot {\overset{-}{\sigma }}_{i}\nabla u_{i}dS=\int_{\Omega_{i}^{\Delta }}\nabla \cdot {\overset{-}{\sigma }}_{i}\nabla u_{i}dV, \end{array}$$

where $\Omega_{i}^{\Delta }$ is the intracellular space contained in the tissue block and $\partial \Omega_{i}^{\Delta }$ is the boundary of the intracellular space contained in the tissue block. This boundary can be separated into the boundary between the intracellular space and the extracellular space contained in the tissue block, i.e. the cell membrane, and the intracellular part of the outer boundary of the tissue block in each spatial direction. Rewriting the surface integral, we obtain


(20) $$\begin{array}{l} \;\int_{A_{i}^{x,+}}{\text{n}}_{i}\cdot {\overset{-}{\sigma }}_{i}\nabla u_{i}dS+\int_{A_{i}^{x,-}}{\text{n}}_{i}\cdot {\overset{-}{\sigma }}_{i}\nabla u_{i}dS\\ +\int_{A_{i}^{y,+}}{\text{n}}_{i}\cdot {\overset{-}{\sigma }}_{i}\nabla u_{i}dS+\int_{A_{i}^{y,-}}{\text{n}}_{i}\cdot {\overset{-}{\sigma }}_{i}\nabla u_{i}dS\\ +\int_{A_{i}^{z,+}}{\text{n}}_{i}\cdot {\overset{-}{\sigma }}_{i}\nabla u_{i}dS+\int_{A_{i}^{z,-}}{\text{n}}_{i}\cdot {\overset{-}{\sigma }}_{i}\nabla u_{i}dS\\ +\int_{\Gamma_{\Delta }}{\text{n}}_{i}\cdot {\overset{-}{\sigma }}_{i}\nabla u_{i}dS=\int_{\Omega_{i}^{\Delta }}\nabla \cdot {\overset{-}{\sigma }}_{i}\nabla u_{i}dV, \end{array}$$

where $A_{i}^{x,+}$ is the intracellular part of the boundary of the tissue block in the positive *x*-direction, $A_{i}^{x,-}$ is the intracellular part of the boundary of the tissue block in the negative *x*-direction, and the surfaces $A_{i}^{y,+}$, $A_{i}^{y,-}$, $A_{i}^{z,+}$, and $A_{i}^{z,-}$ are defined similarly for the intracellular part of the boundaries of the tissue block in the *y*- and *z*-directions. Furthermore, Γ_Δ_ is the membrane contained in the tissue block.

By applying the divergence theorem and similar definitions for the extracellular space, we likewise obtain


(21) $$\begin{array}{l} \;\int_{A_{e}^{x,+}}{\text{n}}_{e}\cdot \sigma_{e}\nabla u_{e}dS+\int_{A_{e}^{x,-}}{\text{n}}_{e}\cdot \sigma_{e}\nabla u_{e}dS\\ +\int_{A_{e}^{y,+}}{\text{n}}_{e}\cdot \sigma_{e}\nabla u_{e}dS+\int_{A_{e}^{y,-}}{\text{n}}_{e}\cdot \sigma_{e}\nabla u_{e}dS\\ +\int_{A_{e}^{z,+}}{\text{n}}_{e}\cdot \sigma_{e}\nabla u_{e}dS+\int_{A_{e}^{z,-}}{\text{n}}_{e}\cdot \sigma_{e}\nabla u_{e}dS\\ +\int_{\Gamma_{\Delta }}{\text{n}}_{e}\cdot \sigma_{e}\nabla u_{e}dS=\int_{\Omega_{e}^{\Delta }}\nabla \cdot \sigma_{e}\nabla u_{e}dV. \end{array}$$

##### Step 3: Applying the EMI Model Equations (12–(14)

By inserting Equations (12) and (13) into Equations (20) and (21), we find that the right hand sides of Equations (20) and (21) are zero. Moreover, by inserting Equation (14), we get


(22) $$\begin{array}{l} \;\int_{A_{i}^{x,+}}{\text{n}}_{i}\cdot {\overset{-}{\sigma }}_{i}\nabla u_{i}dS+\int_{A_{i}^{x,-}}\;{\text{n}}_{i}\cdot {\overset{-}{\sigma }}_{i}\nabla u_{i}dS\\ +\int_{A_{i}^{y,+}}{\text{n}}_{i}\cdot {\overset{-}{\sigma }}_{i}\nabla u_{i}dS+\int_{A_{i}^{y,-}}{\text{n}}_{i}\cdot {\overset{-}{\sigma }}_{i}\nabla u_{i}dS\\ +\int_{A_{i}^{z,+}}{\text{n}}_{i}\cdot {\overset{-}{\sigma }}_{i}\nabla u_{i}dS+\int_{A_{i}^{z,-}}{\text{n}}_{i}\cdot {\overset{-}{\sigma }}_{i}\nabla u_{i}dS-\int_{\Gamma_{\Delta }}I_{m}dS=0 \end{array}$$

for the intracellular part, and


(23) $$\begin{array}{l} \;\int_{A_{e}^{x,+}}{\text{n}}_{e}\cdot \sigma_{e}\nabla u_{e}dS+\int_{A_{e}^{x,-}}{\text{n}}_{e}\cdot \sigma_{e}\nabla u_{e}dS\\ +\int_{A_{e}^{y,+}}{\text{n}}_{e}\cdot \sigma_{e}\nabla u_{e}dS+\int_{A_{e}^{y,-}}{\text{n}}_{e}\cdot \sigma_{e}\nabla u_{e}dS\\ +\int_{A_{e}^{z,+}}{\text{n}}_{e}\cdot \sigma_{e}\nabla u_{e}dS+\int_{A_{e}^{z,-}}{\text{n}}_{e}\cdot \sigma_{e}\nabla u_{e}dS+\int_{\Gamma_{\Delta }}I_{m}dS=0 \end{array}$$

for the extracellular part.

##### Step 4: Extending the Variables and Parameters to Be Defined Everywhere

In order to avoid having to represent the detailed geometry of the cell tissue, we now define some new variables *U*_*i*_, *U*_*e*_, and *V* that each are defined in the entire domain Ω = Ω_*i*_∪Ω_*e*_, and thus also in the entire tissue block, Δ. We want these variables to fulfill the integral conditions specified in Equations (22) and (23). In addition, we assume that the definitions of the membrane potential and *I*_*m*_ specified in Equations (15) and (16) apply in the entire domain. In other words, in an arbitrary tissue block, Δ, of Ω, we seek solutions *U*_*i*_, *U*_*e*_, and *V* such that


(24) $$\begin{array}{l} \;\int_{A_{i}^{x,+}}\text{n}\cdot {\overset{-}{\sigma }}_{i}\nabla U_{i}dS+\int_{A_{i}^{x,-}}\;\text{n}\cdot {\overset{-}{\sigma }}_{i}\nabla U_{i}dS\\ +\int_{A_{i}^{y,+}}\text{n}\cdot {\overset{-}{\sigma }}_{i}\nabla U_{i}dS+\int_{A_{i}^{y,-}}\text{n}\cdot {\overset{-}{\sigma }}_{i}\nabla U_{i}dS\\ +\int_{A_{i}^{z,+}}\text{n}\cdot {\overset{-}{\sigma }}_{i}\nabla U_{i}dS+\int_{A_{i}^{z,-}}\text{n}\cdot {\overset{-}{\sigma }}_{i}\nabla U_{i}dS-\int_{\Gamma_{\Delta }}I_{m}dS=0, \end{array}$$

(25) $$\begin{array}{l} \;\int_{A_{e}^{x,+}}\text{n}\cdot \sigma_{e}\nabla U_{e}dS+\int_{A_{e}^{x,-}}\;\text{n}\cdot \sigma_{e}\nabla U_{e}dS\\ +\int_{A_{e}^{y,+}}\text{n}\cdot \sigma_{e}\nabla U_{e}dS+\int_{A_{e}^{y,-}}\text{n}\cdot \sigma_{e}\nabla U_{e}dS\\ +\int_{A_{e}^{z,+}}\text{n}\cdot \sigma_{e}\nabla U_{e}dS+\int_{A_{e}^{z,-}}\text{n}\cdot \sigma_{e}\nabla U_{e}dS+\int_{\Gamma_{\Delta }}I_{m}dS=0, \end{array}$$

(26) $$\begin{array}{l} V=U_{i}-U_{e}, \end{array}$$

(27) $$\begin{array}{l} I_{m}=C_{m}V_{t}+I_{\text{ion}}. \end{array}$$

Here, **n** is the outward pointing normal vector of the tissue block, and ${\overset{-}{\sigma }}_{i}$, σ_*e*_, *C*_*m*_, *I*_*m*_ and *I*_ion_ have been extended to be defined in the entire domain.

##### Step 5: Approximate the Surface Integrals

Since *I*_*m*_ is now defined in the entire tissue block, and not just on the membrane, the surface integral over the membrane can be approximated by


(28) $$\begin{array}{l} \int_{\Gamma_{\Delta }}I_{m}dS\approx \int_{\Delta }\chi I_{m}dV, \end{array}$$

where Δ is the entire tissue block and χ is the membrane surface to volume ratio, i.e., the surface area of the membrane contained in Δ divided by the volume of Δ.

In addition, the integrals in Equations (24) and (25) over the outer boundary of the tissue block is separated into the intracellular and extracellular parts of the tissue block, and in this step of the derivation, we wish to approximate these integrals to be defined over the entire tissue block boundaries. In order to do this, we apply the approximation


(29) $$\begin{array}{l} \int_{A_{i}^{x,+}}\text{n}\cdot {\overset{-}{\sigma }}_{i}\nabla U_{i}dS\approx {Ā}_{i}^{x}\int_{A^{x,+}}\text{n}\cdot {\overset{-}{\sigma }}_{i}\nabla U_{i}dS, \end{array}$$

and similar approximations for the remaining surfaces. Here, ${Ā}_{i}^{x}$ is the average fraction of the cross-sectional area of the tissue block perpendicular to the *x*-direction that is occupied by the intracellular space and *A*^*x*,+^ is the entire boundary of the tissue block in the positive *x*-direction. Inserting this type of approximation in all the integrals over the outer boundaries of the tissue block, Equations (24) and (25) can be approximated as


(30) $$\begin{array}{l} \;\int_{A^{x,+}}\text{n}\cdot {Ā}_{i}^{x}{\overset{-}{\sigma }}_{i}\nabla U_{i}dS+\int_{A^{x,-}}\;\text{n}\cdot {Ā}_{i}^{x}{\overset{-}{\sigma }}_{i}\nabla U_{i}dS\\ +\int_{A^{y,+}}\text{n}\cdot {Ā}_{i}^{y}{\overset{-}{\sigma }}_{i}\nabla U_{i}dS+\int_{A^{y,-}}\text{n}\cdot {Ā}_{i}^{y}{\overset{-}{\sigma }}_{i}\nabla U_{i}dS\\ +\int_{A^{z,+}}\text{n}\cdot {Ā}_{i}^{z}{\overset{-}{\sigma }}_{i}\nabla U_{i}dS+\int_{A^{z,-}}\text{n}\cdot {Ā}_{i}^{z}{\overset{-}{\sigma }}_{i}\nabla U_{i}dS-\int_{\Delta }\chi I_{m}dV=0, \end{array}$$

(31) $$\begin{array}{l} \;\int_{A^{x,+}}\text{n}\cdot {Ā}_{e}^{x}\sigma_{e}\nabla U_{e}dS+\int_{A^{x,-}}\;\text{n}\cdot {Ā}_{e}^{x}\sigma_{e}\nabla U_{e}dS\\ +\int_{A^{y,+}}\text{n}\cdot {Ā}_{e}^{y}\sigma_{e}\nabla U_{e}dS+\int_{A^{y,-}}\text{n}\cdot {Ā}_{e}^{y}\sigma_{e}\nabla U_{e}dS\\ +\int_{A^{z,+}}\text{n}\cdot {Ā}_{e}^{z}\sigma_{e}\nabla U_{e}dS+\int_{A^{z,-}}\text{n}\cdot {Ā}_{e}^{z}\sigma_{e}\nabla U_{e}dS+\int_{\Delta }\chi I_{m}dV=0. \end{array}$$

Furthermore, we may define a set of scaled bidomain conductivities,


(32) $$\begin{array}{l} {\tilde{\sigma }}_{i}^{x}={Ā}_{i}^{x}{\overset{-}{\sigma }}_{i}^{x},\;{\tilde{\sigma }}_{i}^{y}={Ā}_{i}^{y}{\overset{-}{\sigma }}_{i}^{y},\;{\tilde{\sigma }}_{i}^{z}={Ā}_{i}^{z}{\overset{-}{\sigma }}_{i}^{z}, \end{array}$$

(33) $$\begin{array}{l} {\tilde{\sigma }}_{e}^{x}={Ā}_{e}^{x}\sigma_{e}^{x},\;{\tilde{\sigma }}_{e}^{y}={Ā}_{e}^{y}\sigma_{e}^{y},\;{\tilde{\sigma }}_{e}^{z}={Ā}_{e}^{z}\sigma_{e}^{z}, \end{array}$$

where ${\overset{-}{\sigma }}_{i}^{x}$, ${\overset{-}{\sigma }}_{i}^{y}$ and ${\overset{-}{\sigma }}_{i}^{z}$ and $\sigma_{e}^{x}$, $\sigma_{e}^{y}$ and $\sigma_{e}^{z}$ refer to the possible directional dependence of ${\overset{-}{\sigma }}_{i}$ and σ_*e*_. We also define the associated bidomain conductivity tensors,


(34) $$\begin{array}{l} M_{i}=\left(\begin{array}{ccc} {\tilde{\sigma }}_{i}^{x} & 0 & 0\\ 0 & {\tilde{\sigma }}_{i}^{y} & 0\\ 0 & 0 & {\tilde{\sigma }}_{i}^{z} \end{array}\right),\;M_{e}=\left(\begin{array}{ccc} {\tilde{\sigma }}_{e}^{x} & 0 & 0\\ 0 & {\tilde{\sigma }}_{e}^{y} & 0\\ 0 & 0 & {\tilde{\sigma }}_{e}^{z} \end{array}\right). \end{array}$$

By introducing these tensors, Equations (30) and (31) can be rewritten as


(35) $$\begin{array}{l} \int_{\partial \Delta }\text{n}\cdot \left(M_{i}\nabla U_{i}\right)dS-\int_{\Delta }\chi I_{m}dV=0, \end{array}$$

(36) $$\begin{array}{l} \int_{\partial \Delta }\text{n}\cdot \left(M_{e}\nabla U_{e}\right)dS+\int_{\Delta }\chi I_{m}dV=0, \end{array}$$

where ∂Δ represents the entire outer surface of the tissue block.

##### Step 6: Reapply the Divergence Theorem for the New Variables

We may now reapply the divergence theorem for the newly defined variables *U*_*i*_ and *U*_*e*_ defined in the entire tissue block. This yields


(37) $$\begin{array}{l} \int_{\Delta }\nabla \cdot \left(M_{i}\nabla U_{i}\right)dV-\int_{\Delta }\chi I_{m}dV=0, \end{array}$$

(38) $$\begin{array}{l} \int_{\Delta }\nabla \cdot \left(M_{e}\nabla U_{e}\right)dV+\int_{\Delta }\chi I_{m}dV=0. \end{array}$$

We also note that the volume Δ was chosen arbitrarily. Therefore, the more general relation


(39) $$\begin{array}{l} \nabla \cdot \left(M_{i}\nabla U_{i}\right)-\chi I_{m}=0, \end{array}$$

(40) $$\begin{array}{l} \nabla \cdot \left(M_{e}\nabla U_{e}\right)+\chi I_{m}=0, \end{array}$$

holds.

##### Step 7: Rearranging the Terms and Inserting Equations (26) and (27)

By rearranging Equation (39) and adding Equations (39) and (40), these equations may be rewritten as


(41) $$\begin{array}{l} \nabla \cdot \left(M_{i}\nabla U_{i}\right)=\chi I_{m}, \end{array}$$

(42) $$\begin{array}{l} \nabla \cdot \left(M_{i}\nabla U_{i}\right)+\nabla \cdot \left(M_{e}\nabla U_{e}\right)=0. \end{array}$$

Finally, by inserting Equations (26) and (27), we obtain the bidomain model equations


(43) $$\begin{array}{l} \nabla \cdot \left(M_{i}\nabla V\right)+\nabla \cdot \left(M_{i}\nabla U_{e}\right)=\chi \left(C_{m}\frac{\partial V}{\partial t}+I_{\text{ion}}\right), \end{array}$$

(44) $$\begin{array}{l} \nabla \cdot \left(M_{i}\nabla V\right)+\nabla \cdot \left(\left(M_{i}+M_{e}\right)\nabla U_{e}\right)=0, \end{array}$$

where we recall that *M*_*i*_ and *M*_*e*_ are intracellular and extracellular conductivity tensors (in mS/cm) defined in Equations (32)–(34), χ is the membrane surface to volume ratio (in cm^−1^), *C*_*m*_ is the specific membrane capacitance (in μF/cm^2^), *I*_ion_ is the current density through ion channels, pumps and exchangers on the cell membrane (in μA/cm^2^) and *V* and *U*_*e*_ (in mV) are the bidomain model membrane and extracellular potentials, respectively, defined in the entire domain. Furthermore, the intracellular potential (in mV) may be computed by


(45) $$\begin{array}{l} U_{i}=V+U_{e}. \end{array}$$

In addition, the boundary conditions


(46) $$\begin{array}{l} \;U_{e}=0\text{ at }\partial \Omega^{D}, \end{array}$$

(47) $$\begin{array}{l} \frac{\partial U_{e}}{\partial \text{n}}=0\text{ at }\partial \Omega^{N}, \end{array}$$

are assumed to hold at the boundary of the domain where Ω^*D*^ coincides with the EMI model boundary $\Omega_{e}^{D}$, and Ω^*N*^ coincides with the EMI model boundary $\Omega_{e}^{N}.$

### 2.3. Expressions for the Bidomain Model Parameters

The bidomain model as derived above introduces a set of new parameters, namely the conductivity tensors, *M*_*i*_ and *M*_*e*_, and the surface to volume ratio, χ. Considering their definitions, values for these parameters may be derived from the geometry and parameters of the EMI model. In this subsection, we suggest an approach for making these definitions by considering an EMI model mesh of a volume Ω, containing an intracellular volume, Ω_*i*_, and extracellular volume, Ω_*e*_, a surface for the cell membranes, Γ, and a collection of surfaces for the intercalated discs, Γ_*g*_. For simplicity, we assume that the value of all the EMI model parameters and the tissue geometry do not vary in different parts of the domain, so that the bidomain model parameters can be treated as constants throughout the domain. In addition, we assume that the total domain Ω = Ω_*i*_∪Ω_*e*_ is shaped as a rectangular cuboid with lengths *L*_*x*_, *L*_*y*_ and *L*_*y*_ in the *x*-, *y*- and *z*-directions, respectively. An alternative approach for setting up the bidomain model conductivities from the EMI model parameters and a simplified tissue geometry is presented in Henriquez and Ying (2021).

#### 2.3.1. Surface to Volume Ratio, χ

In order to compute the surface to volume ratio from an EMI model mesh, we may simply compute


(48) $$\begin{array}{l} A_{\Gamma ,\Omega }=\int_{\Gamma }1\;dS, \end{array}$$

(49) $$\begin{array}{l} V_{\Omega }=\int_{\Omega }1\;dS, \end{array}$$

where *A*_Γ,Ω_ represents the total membrane area in the domain and *V*_Ω_ represents the volume of the domain. Assuming an even distribution of cells throughout the domain, the surface to volume ratio can then be defined as


(50) $$\begin{array}{l} \chi =\frac{A_{\Gamma ,\Omega }}{V_{\Omega }}. \end{array}$$

#### 2.3.2. Average Cross-Sectional Area Fractions

We first consider the average intracellular fraction of the cross-sectional area perpendicular to the *x*-axis, ${Ā}_{i}^{x}$. Let *A*^*x*^(*x*) be the cross-sectional area of Ω perpendicular to the *x*-axis, and let $A_{i}^{x}\left(x\right)$ be the fraction belonging to Ω_*i*_. Then


(51) $$\begin{array}{l} V_{\Omega_{i}}=\int_{0}^{L_{x}}A_{i}^{x}\left(x\right)A^{x}\left(x\right)dx={Ā}_{i}^{x}\int_{0}^{L_{x}}A^{x}\left(x\right)dx={Ā}_{i}^{x}V_{\Omega }. \end{array}$$

Hence,


(52) $$\begin{array}{l} {Ā}_{i}^{x}=\frac{V_{\Omega_{i}}}{V_{\Omega }}. \end{array}$$

Similar arguments yield


(53) $$\begin{array}{l} {Ā}_{i}^{x}={Ā}_{i}^{y}={Ā}_{i}^{z}=\frac{V_{\Omega_{i}}}{V_{\Omega }}, \end{array}$$

(54) $$\begin{array}{l} {Ā}_{e}^{x}={Ā}_{e}^{y}={Ā}_{e}^{z}=\frac{V_{\Omega_{e}}}{V_{\Omega }}=\left(1-\frac{V_{\Omega_{i}}}{V_{\Omega }}\right). \end{array}$$

Here, *V*_Ω_*i*__ can be computed from the EMI model mesh as


(55) $$\begin{array}{l} V_{\Omega_{i}}=\int_{\Omega_{i}}1\;dS. \end{array}$$

#### 2.3.3. Average Intracellular Conductivity

As described above, we wish to define an average conductivity ${\overset{-}{\sigma }}_{i}$ such that the simplified EMI model (Equations 12–18) is a good approximation of the full EMI model (Equations 1–11). In particular, we wish to find a ${\overset{-}{\sigma }}_{i}$ such that the total intracellular resistance of the simplified model is close to the total intracellular resistance of the full model. To simplify this argument, we assume that there is no capacitive current across the intercalated discs, i.e. that the current between two cells is given by *I*_gap_ (see Equation 11).

We start by considering the total resistance in the *x*-direction of the domain. In the full EMI model (Equations 1–11) with the capacitive current set to zero, this is given by the sum of the resistance over the purely intracellular space ($R_{c}^{x}$) and the resistance over the gap junctions ($R_{j}^{x}$) (Shaw and Rudy, 1997):


(56) $$\begin{array}{l} R_{i}^{x}=R_{c}^{x}+R_{j}^{x}. \end{array}$$

The total resistance in the purely intracellular space is given by (Plonsey and Barr, 2007)


(57) $$\begin{array}{l} R_{c}^{x}=\frac{L_{x}}{\sigma_{i}{Ā}_{i}^{x}L_{y}L_{z}}, \end{array}$$

where ${Ā}_{i}^{x}L_{y}L_{z}$ is the average intracellular cross-sectional area of the domain perpendicular to the *x*-direction. Assuming that the cells are organized as a regular grid in the *x*-, *y*- and *z*-directions, the total resistance through gap junctions in the *x*-direction is given by


(58) $$\begin{array}{l} R_{j}^{x}=\frac{\left(N_{x}-1\right)R_{g}}{N_{y}N_{z}A_{j}^{x}}, \end{array}$$

where *R*_*g*_ is the specific gap junction resistance (in kΩcm^2^), as it appears in the full EMI model, $A_{j}^{x}$ is the area of a single intercalated disc perpendicular to the *x*-direction and *N*_*x*_, *N*_*y*_, and *N*_*z*_ are the number of cells in the *x*-, *y*-, and *z*-directions, respectively. Thus, *N*_*x*_ − 1 is the number of intercalated disc collections along the length of the domain in the *x*-direction, *N*_*y*_*N*_*z*_ is the number of intercalated disc for each such collection, and $N_{y}N_{z}A_{j}^{x}$ is the total cross-sectional area of each of the intercalated disc collections.

In the simplified model (Equations 12–18), the total resistance is given by (Plonsey and Barr, 2007)


(59) $$\begin{array}{l} R_{i}^{x}=\frac{L_{x}}{{\overset{-}{\sigma }}_{i}^{x}{Ā}_{i}^{x}L_{y}L_{z}}. \end{array}$$

Therefore, in order for the total resistance to be the same in the two formulations, we wish ${\overset{-}{\sigma }}_{i}^{x}$ to satisfy


(60) $$\begin{array}{l} \frac{L_{x}}{{\overset{-}{\sigma }}_{i}^{x}{Ā}_{i}^{x}L_{y}L_{z}}=\frac{L_{x}}{\sigma_{i}{Ā}_{i}^{x}L_{y}L_{z}}+\frac{\left(N_{x}-1\right)R_{g}}{N_{y}N_{z}A_{j}^{x}}, \end{array}$$

which yields


(61) $$\begin{array}{l} {\overset{-}{\sigma_{i}}}^{x}=\frac{\sigma_{i}}{1+\frac{\sigma_{i}R_{g}\left(N_{x}-1\right){Ā}_{i}^{x}L_{y}L_{z}}{L_{x}N_{y}N_{z}A_{j}^{x}}}. \end{array}$$

From the EMI model mesh, we may compute


(62) $$\begin{array}{l} A_{j,\Omega }^{x}=\int_{\Gamma_{g}^{x}}1\;dS, \end{array}$$

as the total area of all intercalated discs perpendicular to the *x*-direction, $\Gamma_{g}^{x}$. Since $A_{j}^{x}$ is defined as the area of a single intercalated disc perpendicular to the *x*-direction, we note that


(63) $$\begin{array}{l} A_{j}^{x}=\frac{A_{j,\Omega }^{x}}{\left(N_{x}-1\right)N_{y}N_{z}}, \end{array}$$

where (*N*_*x*_ − 1)*N*_*y*_*N*_*z*_ is the total number of intercalated discs in the *x*-direction. Inserting Equations (63) into Equation (61) yields


(64) $$\begin{array}{l} {\overset{-}{\sigma }}_{i}^{x}=\frac{\sigma_{i}}{1+\frac{{\left(N_{x}-1\right)}^{2}{Ā}_{i}^{x}L_{y}L_{z}\sigma_{i}R_{g}}{L_{x}A_{j,\Omega }^{x}}}. \end{array}$$

We also note that from Equation (52), we have that


(65) $$\begin{array}{l} {Ā}_{i}^{x}=\frac{V_{\Omega_{i}}}{V_{\Omega }}=\frac{V_{\Omega_{i}}}{L_{x}L_{y}L_{z}}\;\Rightarrow \;{Ā}_{i}^{x}L_{y}L_{z}=\frac{V_{\Omega_{i}}}{L_{x}}, \end{array}$$

and inserting this into Equation (64), we obtain


(66) $$\begin{array}{l} {\overset{-}{\sigma }}_{i}^{x}=\frac{\sigma_{i}}{1+\frac{\sigma_{i}R_{g}V_{\Omega_{i}}}{\delta_{x}^{2}A_{j,\Omega }^{x}}}. \end{array}$$

where δ_*x*_ = *L*_*x*_/(*N*_*x*_ − 1). Similar arguments for the *y*- and *z*-directions result in


(67) $$\begin{array}{l} {\overset{-}{\sigma }}_{i}^{y}=\frac{\sigma_{i}}{1+\frac{\sigma_{i}R_{g}V_{\Omega_{i}}}{\delta_{y}^{2}A_{j,\Omega }^{y}}}, \end{array}$$

(68) $$\begin{array}{l} {\overset{-}{\sigma }}_{i}^{z}=\frac{\sigma_{i}}{1+\frac{\sigma_{i}R_{g}V_{\Omega_{i}}}{\delta_{z}^{2}A_{j,\Omega }^{z}}}. \end{array}$$

Since


(69) $$\begin{array}{l} \frac{{\overset{-}{\sigma }}_{i}^{x}}{{\overset{-}{\sigma }}_{i}^{y}}=\frac{1+\frac{\sigma_{i}R_{g}V_{\Omega_{i}}}{\delta_{y}^{2}A_{j,\Omega }^{y}}}{1+\frac{\sigma_{i}R_{g}V_{\Omega_{i}}}{\delta_{x}^{2}A_{j,\Omega }^{x}}}, \end{array}$$

the anisotropy is governed by the difference between $\delta_{x}^{2}A_{j,\Omega }^{x}$ and $\delta_{y}^{2}A_{j,\Omega }^{y},$ and similar for the other combination of axes.

#### 2.3.4. Intracellular Conductivity Tensor

Inserting Equations (52) and (66)–(68) into Equation (32), we get


(70) $$\begin{array}{l} {\tilde{\sigma }}_{i}^{x}={Ā}_{i}^{x}{\overset{-}{\sigma }}_{i}^{x}=\frac{V_{\Omega_{i}}}{V_{\Omega }}\frac{\sigma_{i}}{1+\frac{\sigma_{i}{\left(N_{x}-1\right)}^{2}R_{g}V_{\Omega_{i}}}{L_{x}^{2}A_{j,\Omega }^{x}}}, \end{array}$$

(71) $$\begin{array}{l} {\tilde{\sigma }}_{i}^{y}={Ā}_{i}^{y}{\overset{-}{\sigma }}_{i}^{y}=\frac{V_{\Omega_{i}}}{V_{\Omega }}\frac{\sigma_{i}}{1+\frac{\sigma_{i}{\left(N_{y}-1\right)}^{2}R_{g}V_{\Omega_{i}}}{L_{y}^{2}A_{j,\Omega }^{y}}}, \end{array}$$

(72) $$\begin{array}{l} {\tilde{\sigma }}_{i}^{z}={Ā}_{i}^{z}{\overset{-}{\sigma }}_{i}^{z}=\frac{V_{\Omega_{i}}}{V_{\Omega }}\frac{\sigma_{i}}{1+\frac{\sigma_{i}{\left(N_{z}-1\right)}^{2}R_{g}V_{\Omega_{i}}}{L_{z}^{2}A_{j,\Omega }^{z}}}. \end{array}$$

#### 2.3.5. Extracellular Conductivity Tensor

The extracellular conductivity tensor can be found directly from the cross-sectional area fractions and we get


(73) $$\begin{array}{l} {\tilde{\sigma }}_{e}^{x}={\tilde{\sigma }}_{e}^{y}={\tilde{\sigma }}_{e}^{z}=\frac{V_{\Omega_{e}}}{V_{\Omega }}\sigma_{e}=\left(1-\frac{V_{\Omega_{i}}}{V_{\Omega }}\right)\sigma_{e}. \end{array}$$

## 3. Results

In order to compare the EMI model with the homogenized bidomain model, we set up a few example applications and perform numerical simulations of the two models. Note here that all EMI model simulations are performed in three dimensions (3D), whereas the bidomain model simulations are performed in two dimensions (2D) or one dimension (1D).

### 3.1. Simulation Set-Up

In our numerical simulations of the EMI model, we consider collections of cells shaped as cylinders with a slightly varying diameter. In all simulations, except for the ones where the cell length is varied and is explicitly specified, each cell is 120 μm long (in the *x*-direction) and has a radius varying from 6 μm at the cell ends to 7 μm at the center of the cell (see Figure 1). We let the distance from the boundary of the extracellular space to the cell collection be 2 μm in all spatial directions. The parameter values used in the simulations are specified in Table 1. The parameters used in the bidomain model are computed from the EMI model parameters and mesh as described in section 2.3.

**Table 1:** Default parameter values used in the simulations.

**Parameter**	**Value**	**Parameter**	**Value**
σ_*i*_	4 mS/cm	σ_*e*_	20 mS/cm
*C*_*m*_	1 μF/cm^2^	*C*_*g*_	0.5 μF/cm^2^
*R*_*g*_	0.0015 kΩcm^2^		

*The bidomain model parameters M_i_, M_e_ and χ are computed from the EMI model parameters and mesh as described in section 2.3*.

All EMI model simulations are performed in 3D. However, for our example test cases with a 1D strand of cells and a 2D grid of cells, we use 1D and 2D versions, respectively, of the bidomain model. In the simulations of a 1D strand of cells, we apply homogenous Neumann boundary conditions on the outer boundary of the extracellular domain in the *y*- and *z*-directions and homogenous Dirichlet boundary conditions in the *x*-direction. In the simulations of a 2D grid of cells, we apply homogenous Neumann boundary conditions on the outer boundary of the extracellular domain in the *z*-direction and homogenous Dirichlet boundary conditions in the *x*- and *y*-directions.

### 3.2. Numerical Methods

The EMI model simulations are performed using the operator splitting procedure described in Jæger et al. (2021c,d), the numerical methods applied to (Jæger et al., 2021d) and the MFEM C++ finite element method library (Anderson et al., 2020; MFEM, 2021). For details on the numerical methods applied to solve the EMI model, we refer to Jæger et al. (2021a,c,d). The bidomain model simulations are performed in Matlab using a first-order temporal operator splitting procedure as described in Sundnes et al. (2006), where the ordinary differential part of the equations is solved using forward Euler and the partial differential part of the equations is solved using an implicit finite difference scheme. Unless otherwise specified, we use a time step of Δ*t* = 0.001 ms in the simulations of both models. In the bidomain model simulations, we use a spatial discretization of Δ*x* = Δ*y* = 10 μm, roughly matching the typical edge length in the applied EMI model finite element mesh.

### 3.3. 1D Strand of Cells With a Passive Membrane Model

**Figure 3:**
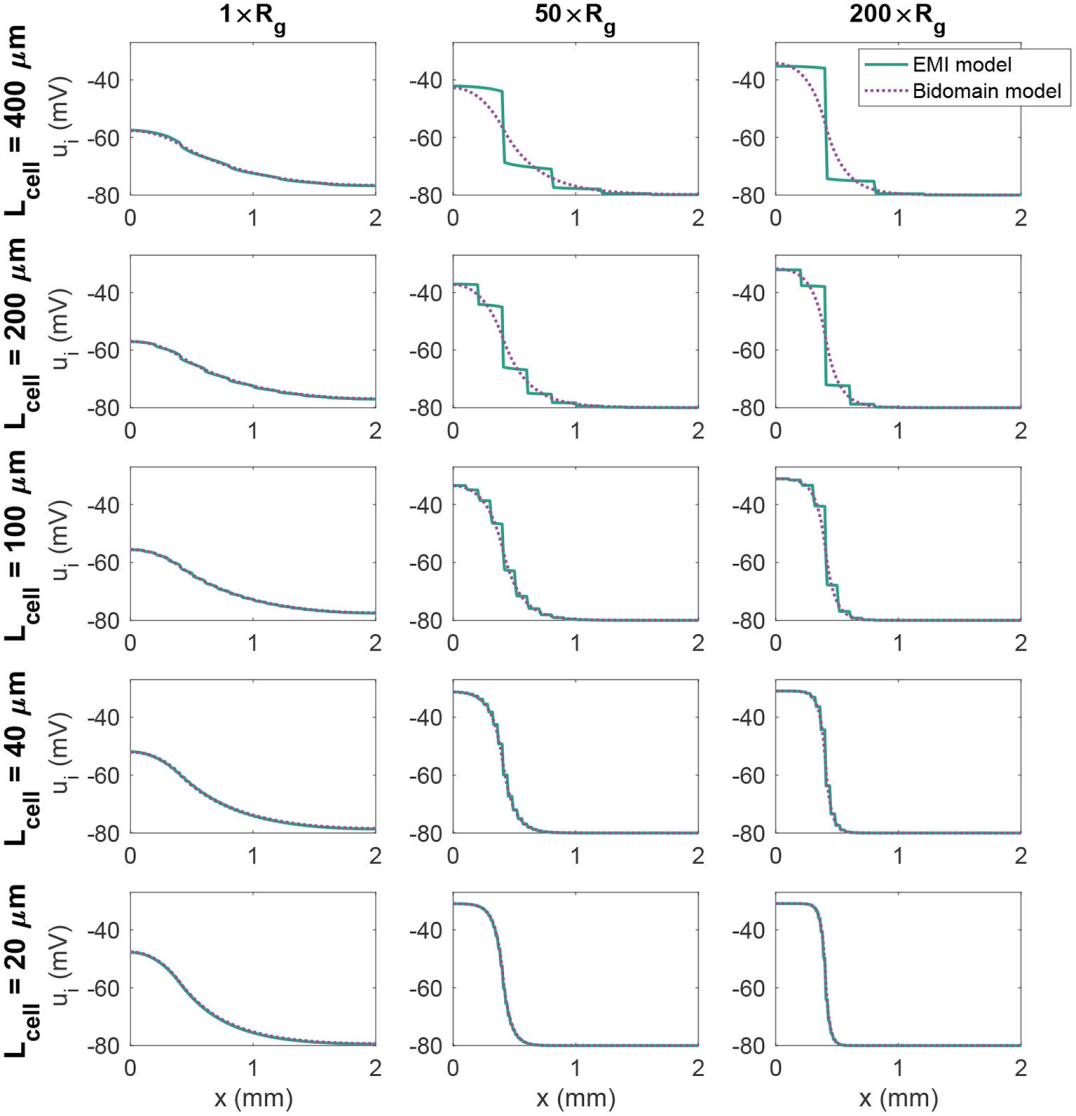
Intracellular potential, *u*_*i*_, at time *t* = 20 ms in EMI model and bidomain model simulations using a passive membrane model (Equation 74) and the default parameter values specified in Table 1, except that the value of *R*_*g*_ is increased by the factor indicated by the column titles. In addition, the cell length (*L*_cell_) is varied as described for each row of plots.

We first consider an example with a 1D strand of cells connected in the longitudinal direction (*x*-direction). The total length of the cell strand is 2 mm and we consider a number of different choices for the length of a single cell (and the associated total number of cells). In addition, we vary the value of the specific gap junction resistance, *R*_*g*_. The membrane dynamics, *I*_ion_, is modeled by a simple passive membrane model


(74) $$\begin{array}{l} I_{\text{ion}}=\frac{1}{R_{m}}\left(v-v_{0}\right), \end{array}$$

where *R*_*m*_ = 5 kΩcm^2^ is the specific membrane resistance and *v*_0_ = −80 mV is the resting membrane potential. We stimulate the first (leftmost) 400 μm of membrane in the *x*-direction by a constant stimulus current of size −10 μA/cm^2^. Figure 3 shows the intracellular potential at time *t* = 20 ms along a line in the *x*-direction in the center of the domain for the EMI model and the associated solution of the bidomain model for a few combinations of cell length and *R*_*g*_ values. We observe that for small cells (lower panels), the solution of the bidomain model is in very good agreement with the results of the EMI model. However, if the cell size is increased, and the gap junction resistance is increased, there is a significant difference between the results of the EMI model and the bidomain model.

### 3.4. 1D Strand of Cells With an Active Membrane Model

Next, we consider an example with a 1D strand of 20 cells of length 120 μm with an active membrane model, modeled by the human left atrial base model from Jæger et al. (2021b). We initiate a traveling wave by stimulating the first 360 μm of cell membrane in the *x*-direction (corresponding to three cardiomyocytes) by a 1 ms long constant stimulus current of size −40 μA/cm^2^. We measure the conduction velocity as the distance between a point *a* in the center of the domain in the *x*-direction and a point *b* located at 4/5 of the total domain length, divided by the difference in time between when the membrane potential in these two points reach a value above −20 mV. Using the default parameter values specified in Table 1, we get a conduction velocity of 50.8 cm/s in the bidomain model simulation. This is close to the value found in the EMI model simulation, which is 53.3 cm/s.

**Figure 4:**
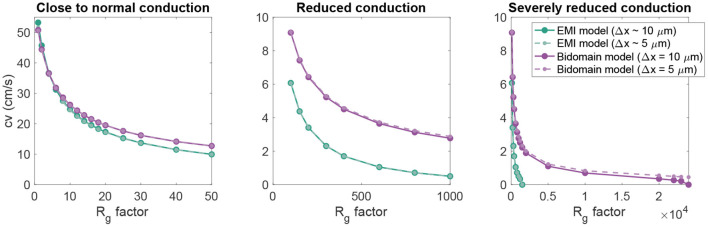
Conduction velocity as *R*_*g*_ is increased in EMI model and bidomain model simulations of a strand of 20 connected cells with an active membrane model (Jæger et al., 2021b). The values on the *x*-axis represent the factor with which the default *R*_*g*_ value in Table 1 is multiplied. The remaining parameter values are as specified in Table 1. Note that the plot is separated into three panels in order to improve the visibility of the data. Note also that we consider two different discretization resolutions in the simulations of each model. In the default case, Δ*t* = 0.001 ms and Δ*x* in the bidomain model and the typical edge length in the EMI model is 10 μm, whereas in the refined case (dashed lines), both the spatial and temporal discretization steps are reduced to half of the default values.

In Figure 4, we further investigate the relationship between the conduction velocity found in the bidomain and EMI model simulations when *R*_*g*_ is increased, representing reduced cell coupling. We consider two different discretization resolutions, the default resolution of Δ*t* = 0.001 ms and Δ*x* ~ 10 μm and a refined resolution of Δ*t* = 0.0005 ms and Δ*x* ~ 5 μm. We observe that for values of *R*_*g*_ relatively close to the default value, the conduction velocities found in simulations of the bidomain and EMI models are very similar. However, when *R*_*g*_ is considerably increased, the difference between the two model formulations appears to be more significant and the conduction velocity is considerably higher in the bidomain model simulations than in the corresponding EMI model simulations. Furthermore, we observe that for the EMI model, conduction is blocked when *R*_*g*_ is increased by a factor larger than about 2,000, whereas for the bidomain model, *R*_*g*_ can be increased by a factor of about 20,000 before conduction is blocked for the default resolution and conduction is not blocked for the considered values of *R*_*g*_ for the refined resolution. In addition, we note that the simulations of refined resolution appears to give very similar conduction velocities as for the default resolution in the EMI model simulations. For the bidomain model simulations, the two resolutions give very similar results for the first range of *R*_*g*_ values, but as the *R*_*g*_ value is severely increased, we can observe a difference between the two resolutions.

### 3.5. 2D Grid of Cells With an Active Membrane Model

**Figure 5:**
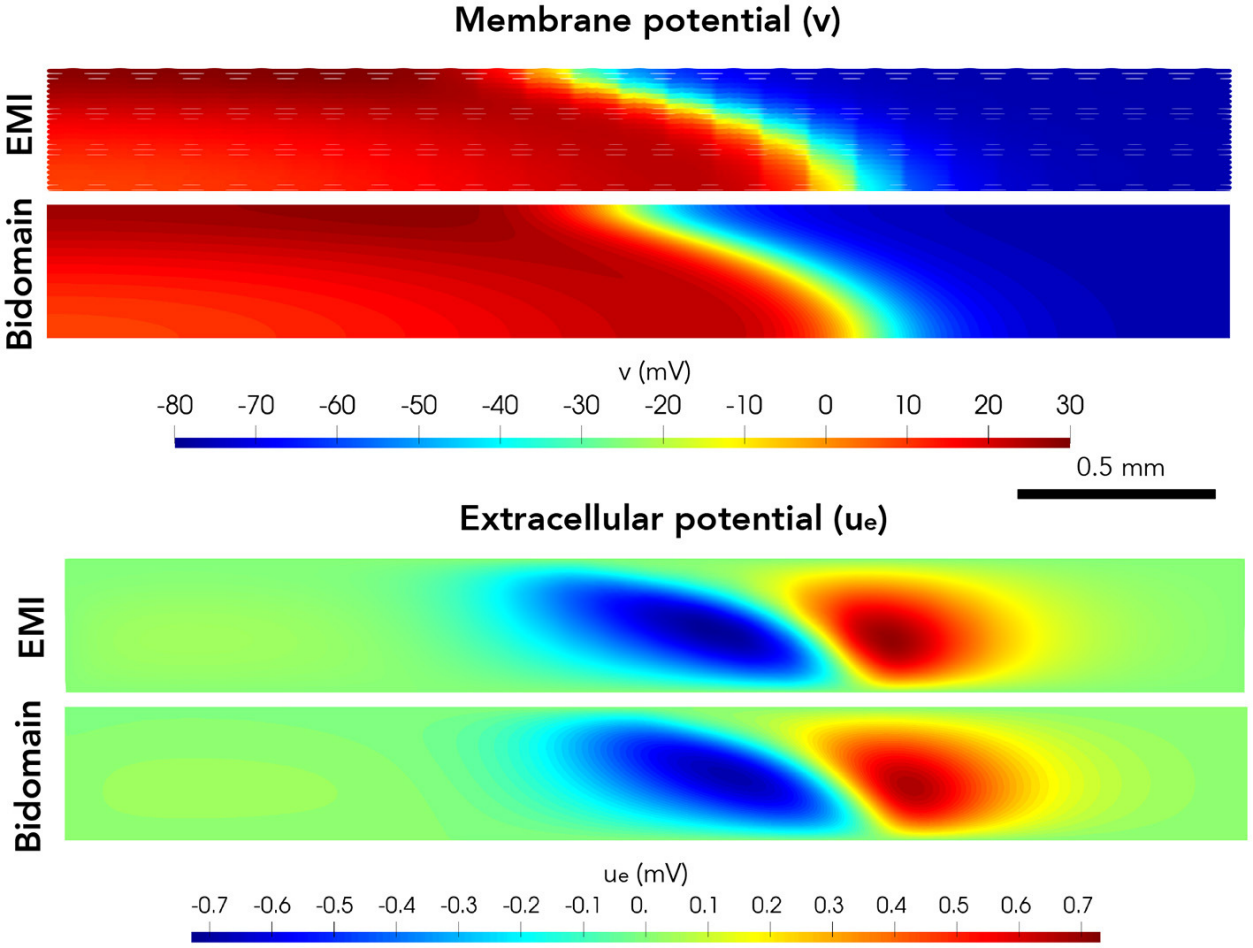
Membrane potential, *v*, and extracellular potential, *u*_*e*_, at time *t* = 5 ms in EMI model and bidomain model simulations using the default parameter values specified in Table 1 and an active membrane model (Jæger et al., 2021b). This simulation required a CPU time of 132 min for the EMI model and 2 min for the bidomain model.

**Figure 6:**
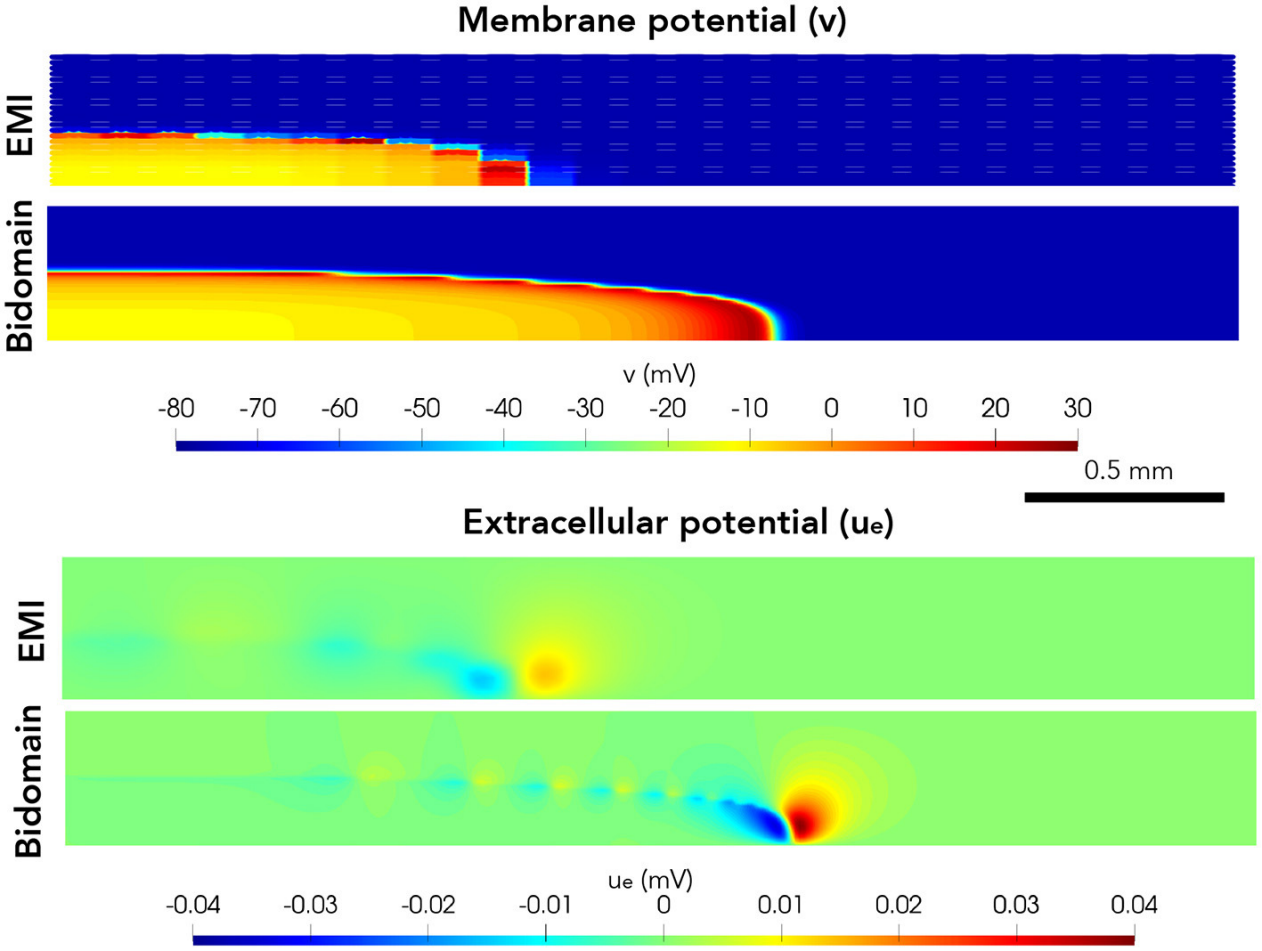
Membrane potential, *v*, and extracellular potential, *u*_*e*_, at time *t* = 20 ms in EMI model and bidomain model simulations using and active membrane model (Jæger et al., 2021b) and the default parameter values specified in Table 1, except that the value of *R*_*g*_ is increased by a factor of 200.

Next, we consider a case of a grid of 25×25 connected cells with the same active membrane model as for the 1D strand simulations. We stimulate the membrane of an area corresponding to the 5×5 cells in the lower left corner by the same stimulation current as in the 1D case. Figure 5 shows the membrane potential, *v*, and the extracellular potential, *u*_*e*_, from the EMI model and bidomain model simulations using the default parameter values specified in Table 1 at time *t* = 5 ms. The solution of the two models appears to be very similar. However, in Figure 6, we have performed a similar simulation where *R*_*g*_ is increased by a factor of 200. We consider the solution at *t* = 20 ms and observe that the traveling excitation wave has clearly traveled faster and reached further in the bidomain model simulation than in the EMI model simulation, consistent with the results of Figure 4.

## 4. Discussion

The bidomain model continues to provide essential insights into cardiac conduction and how the electrochemical dynamics of the heart is affected by blocking ion channels (see, e.g., Zemzemi et al., 2013; Sharifi, 2017), increasing gap junction resistance (see, e.g., Roth, 1988; Bruce et al., 2014), introducing ischemia (see, e.g., Stinstra et al., 2004, 2005; Heidenreich et al., 2012) or performing defibrillation (see, e.g., Skouibine et al., 2000; Trayanova et al., 2006, 2011; Quarteroni et al., 2017). However, as almost any model, its utility is limited by the inherent resolution of the model. It is useful for understanding cardiac conduction at the tissue level, but it cannot be applied for analyses of conduction close to individual cardiomyocytes. Therefore, detailed models representing individual myocytes have been developed.

Here, we show that the bidomain model can be derived directly from the cell-based EMI model. Classically, the bidomain model is derived using elegant homogenization techniques (see Neu and Krassowska, 1993; Henriquez and Ying, 2021). In the derivation presented here, the deviation between the properties of the bidomain model and the EMI model is seen directly as part of the derivation. In short, the advantage of the present derivation is that it is more straightforward to follow and that it gives indications of where the deviations in the results between the two models stem from.

### 4.1. Source of Difference Between EMI and Bidomain Solutions

There are essentially three steps in the derivation of the bidomain model where approximations are introduced and thus, most likely, are responsible for the difference in the solutions of the two models. First; the resistance of the intracellular space and the gap junctions are combined into one common and averaged resistance. Second, the average of a function over a volume is approximated by the average of the same function over the surface of the volume. Third, the average of a function on a surface is approximated by the average of the same function on an extended surface. It is beyond the scope of this paper to perform a detailed analysis of these deviations, but based on these observations, it comes as no surprice that the error becomes smaller when the cell size is reduced.

### 4.2. Differences and Similarities

The EMI model and the bidomain model provide remarkably similar results when the parameters of importance for the conduction velocity are in the normal range. It is safe to claim that the bidomain model represents normal cardiac conduction very well if the scale of interest contains many cells. Certainly, the bidomain model cannot be used to study conduction in the vicinity of individual cells, and it also runs into difficulties for large cells combined with high values of resistance across the gap junctions. It is observed that the bidomain model consistently overestimates the conduction velocity. For normal parameters, the difference is small, but for strongly increased resistance across the gap junctions, the bidomain model significantly overestimates the conduction velocity.

## Data Availability Statement

The original contributions presented in the study are included in the article/supplementary material, further inquiries can be directed to the corresponding author.

## Author Contributions

KH and AT: concept and writing. KH: derivation of models and simulations. AT: definition of test problems. Both authors contributed to the article and approved the submitted version.

## Funding

This project was supported by the Norwegian Research Council through the EMIx project; 324239.

## Conflict of Interest

The authors declare that the research was conducted in the absence of any commercial or financial relationships that could be construed as a potential conflict of interest.

## Publisher's Note

All claims expressed in this article are solely those of the authors and do not necessarily represent those of their affiliated organizations, or those of the publisher, the editors and the reviewers. Any product that may be evaluated in this article, or claim that may be made by its manufacturer, is not guaranteed or endorsed by the publisher.
